# Less advanced disease at initiation of salvage androgen deprivation therapy is associated with decreased mortality following biochemical failure post-salvage radiation therapy

**DOI:** 10.1186/s13014-014-0245-z

**Published:** 2014-11-26

**Authors:** Corey C Foster, William C Jackson, Benjamin C Foster, Skyler B Johnson, Felix Y Feng, Daniel A Hamstra

**Affiliations:** Department of Radiation Oncology, University of Michigan, 1500 E. Medical Center Dr, Ann Arbor, MI 48105 USA; The University of Michigan Medical Center, 1500 East Medical Center Drive, Ann Arbor, MI 48105 USA

**Keywords:** Salvage androgen deprivation therapy, Prostate-specific antigen doubling time, Salvage radiation therapy, Prostate cancer

## Abstract

**Background:**

The optimal clinical context for initiation of salvage androgen deprivation therapy (SADT) following the biochemical recurrence of localized prostate cancer remains controversial. We chose to investigate if disease burden at time of SADT initiation is associated with clinical outcomes following biochemical failure (BF) post-salvage radiation therapy (SRT).

**Methods:**

Medical records of 575 patients receiving SRT at a single institution from 1986–2010 were retrospectively reviewed. Of 250 patients experiencing BF post-SRT, 172 had a calculable prostate-specific antigen doubling time (PSADT) prior to SADT initiation. These patients comprise the analyzed cohort and were divided into four groups based on characteristics at SADT initiation: those with PSADTs >3 months without distant metastasis (DM) (group 1 [less advanced disease], n = 62), those with PSADTs <3 months without DM (group 2 [more advanced disease], n = 28), those with DM (group 3 [more advanced disease], n = 32), and those not receiving SADT during follow-up (group 4, n = 50). Endpoints included prostate cancer-specific mortality (PCSM) and overall mortality (OM). Kaplan-Meier methods were used to estimate survival, and Cox proportional hazards models were used for multivariate analysis.

**Results:**

Median follow-up post-SRT was 7.9 years. Patients starting SADT with more advanced disease were at significantly increased risk for PCSM (hazard ratio [HR]:2.8, 95% confidence interval [CI]: 1.4–5.6, p = 0.005) and OM (HR:1.9, 95% CI: 1.0–3.5, p = 0.04) compared to those receiving SADT with less advanced disease. PCSM and OM did not significantly differ between groups 1 and 4 or groups 2 and 3. Of note, patients in group 4 had very long PSADTs (median = 27.0 months) that were significantly longer than those of group 1 (median = 6.0 months) (p < 0.001). Multivariate analysis including groups 1–3 found a pre-SADT PSADT <3 months to be the most significant predictor of PCSM (HR:4.2, 95% CI: 1.6–11.1, p = 0.004) and the only significant predictor of OM (HR:2.9, 95% CI: 1.3–6.7, p = 0.01).

**Conclusions:**

Less advanced disease at initiation of SADT is associated with decreased PCSM and OM following BF post-SRT; however, observation may be reasonable for patients with very long PSADTs. A PSADT <3 months prior to SADT initiation significantly predicts an increased risk of PCSM and OM in this patient demographic.

## Background

It is estimated that the majority of patients receiving salvage radiation therapy (SRT) for biochemically recurrent prostate cancer post-radical prostatectomy (RP) will not achieve long-term disease control [1]. Eventually, many such patients will once again develop a rising prostate-specific antigen (PSA) after radiotherapy (RT), suggesting the presence of residual local or metastatic disease. Having exhausted their options for local therapy, only systemic treatments remain for these patients with recurrence post-SRT. One common intervention in this setting is the initiation of salvage androgen deprivation therapy (SADT).

While SADT is clearly beneficial for metastatic prostate cancer [2] and initiating SADT while disease burden is low in the setting of advanced disease appears to be clinically advantageous [3], the optimal clinical context for initiation of SADT following the development of biochemical failure (BF) in the absence of metastatic disease remains controversial [4,5]. Much of this controversy stems from the lack of prospective, randomized trials investigating the potential benefits of initiating SADT while disease burden is low for patients experiencing BF alone after local treatment [4,6]. Nonetheless, retrospective studies have consistently suggested that beginning SADT early in the clinical course of recurrent disease post-definitive RT is associated with improved overall survival [7-11]. The purpose of this retrospective analysis is to assess whether less advanced disease at initiation of SADT is similarly associated with improved clinical outcomes following BF post-SRT.

## Methods

### Patient selection

The medical records of 575 patients receiving SRT with or without concurrent androgen deprivation therapy (ADT) at a single institution from 1986 to 2010 were retrospectively reviewed in an institutional review board-approved analysis. SRT was defined as any post-RP RT for a persistently elevated PSA post-RP or for BF post-RP as defined by the American Urologic Association [12]. Of the 575 patients receiving SRT, 250 (43%) went on to experience BF, with BF post-SRT defined as the development of a PSA value 0.2 ng/mL or more above the post-SRT PSA nadir followed by another higher PSA value [1] or any single post-SRT PSA value of 0.5 ng/mL or greater. A total of 172 patients who experienced BF post-SRT had a calculable prostate-specific antigen doubling time (PSADT) prior to starting SADT, and they comprise the cohort used for this analysis. Of these patients, 122 (70%) went on to receive SADT, which was defined as any ADT given after the development of BF or distant metastasis (DM) following SRT. PSADTs were calculated as previously described [13]. A minimum of 3 consecutive PSA values preceding the initiation of SADT was required for the calculation of pre-SADT PSADT, and all available PSA values following BF post-SRT were included in the calculation of PSADTs for patients not receiving SADT during follow-up.

The study was conducted after IRB approval at the University of Michigan Medical Center (Ann Arbor, MI).

### Treatment

Three-dimensional conformal RT or intensity-modulated RT was used for SRT delivery with greater than 95% of patients receiving an SRT dose between 64.0–70.2 Gy. Sixteen patients (9.3%) received whole-pelvic RT. Twenty-five patients (14.5%) received concurrent ADT during SRT. The decision of whether or not to prescribe concurrent ADT with SRT was made on an individual basis after consideration of clinical and pathologic risk factors. The type of SADT given and the timing of SADT initiation following BF post-SRT were at the discretion of the treating physician. When starting SADT, the most frequently prescribed options included a gonadotropin-releasing hormone agonist alone or a gonadotropin-releasing hormone agonist in combination with a non-steroidal antiandrogen. These regimens were given to 39% and 30% of patients receiving SADT, respectively.

### Endpoints

Primary outcomes included prostate cancer-specific mortality (PCSM) and overall mortality (OM). PCSM was defined as any death in the setting of metastatic prostate cancer or castration resistant disease as well as any death otherwise attributed to prostate cancer. OM was defined as death from all causes.

### Statistical analysis

Patients were stratified into four groups based on whether or not SADT was initiated, pre-SADT PSADT, and the presence or absence of DM at the time of SADT initiation. Patients with a PSADT >3 months in the absence of known DM prior to starting SADT were considered to have less advanced disease at SADT initiation. Those with known DM or a PSADT <3 months without DM upon SADT initiation were considered to have more advanced disease at SADT initiation. Pre-SADT PSADT was chosen as a discriminatory clinical characteristic when determining disease burden at initiation of SADT because of the previously described ability of a short post-treatment PSADT to predict poor clinical outcomes following RP [13-16], definitive RT [15,16], and SRT [1,17], implying that patients with shorter PSADTs have more advanced disease. A cut-point of 3 months was chosen for stratification of pre-SADT PSADTs as it is was the closest commonly reported threshold to our median PSADT of 4.4 ng/mL and because a post-treatment PSADT <3 months has been shown to predict PCSM following RP or definitive RT, suggesting patients with PSADTs below this threshold may have a higher disease burden [15,16].

Univariate survival analyses were performed using Kaplan-Meier methods and log-rank tests. The methods of Harrell *et al.* were used to calculate concordance indices (c-indices) with ties being awarded one half their value [18]. Cox proportional hazards models were used for multivariate analysis. Survival was analyzed from the time of BF post-SRT to avoid potential lead-time bias associated with analyzing survival from the start of SADT. All statistical analyses were performed using MedCalc (v 12.7.8, MedCalc Software, Ostend, Belgium).

## Results

### Patient characteristics

The 172 patients in the cohort used for analysis had a median follow-up of 7.9 years (interquartile range [IQR]: 5.7–10.9) following SRT. Sixty-two patients having PSADTs >3 months without DM prior to starting SADT were included in group 1. Group 2 included 28 patients with PSADTs <3 months and no DM before SADT administration while group 3 consisted of 32 patients with known DM preceding initiation of SADT. Fifty-five patients not receiving SADT or other salvage therapies during follow-up comprised group 4. Group 1 was considered to have less advanced disease at initiation of SADT whereas patients in groups 2 and 3 were considered to have more advanced disease at initiation of SADT.

Pre-treatment, treatment, and pathologic characteristics stratified by patient group are displayed in Table 1. Significant differences among the four groups included a lower percentage of patients with Gleason scores of 8–10 in groups 1 and 4 (p = 0.04), a higher percentage of patients with Gleason scores of 2–6 in group 4 (p = 0.04), and a lower percentage of patients with an undetectable PSA nadir post-SRT in group 4 (p = 0.005). Patients in group 4 had a median follow-up post-BF of 3.6 years (range: 0.4–15.3) and notably long PSADTs following BF post-SRT (median = 27.0 months, IQR: 13.6–47.7). The PSADTs of this group were statistically significantly longer than those in groups 1–3 (p < 0.001) and those in group 1 alone (p < 0.001). Additionally, the interval to biochemical failure (IBF) after beginning SRT for group 4 was significantly longer than the IBF for other groups (p < 0.001), and the percentage of patients with a short IBF (defined as <18 months [19]) was significantly lower as well (p < 0.0001). There was no statistically significant difference in the length of IBF or percentage of patients with an IBF <18 months among groups 1–3. Patients in groups 1–3 with a short IBF experienced BF a median of 7.9 months (mean = 9.0) after starting SRT and had a median pre-SADT PSADT of 4.4 months (IQR: 2.4–6.1) while those with a long IBF experienced BF a median of 29.8 months (mean = 33.2) after starting SRT and had a median pre-SADT PSADT of 5.8 months (IQR: 2.7–9.1) (p = 0.04). Other pertinent pathologic characteristics did not differ significantly among the four groups.

**Table 1:** **Pre-treatment, treatment, and pathologic characteristics stratified by patient group**

	**Group 1 (PSADT >3 months, DM absent at start of SADT) (n = 62)**	**Group 2 (PSADT <3 months, DM absent at start of SADT) (n = 28)**	**Group 3 (DM present at start of SADT) (n = 32)**	**Group 4 (No SADT) (n = 50)**	
Age at SRT (y), median (IQR)	65.7 (58.9–70.4)	63.0 (56.6–67.3)	63.6 (57.8–67.6)	62.3 (55.9–67.5)	p = 0.38*
CCMI, median (range) (n = 147)	3.0 (1.0–6.0)	3.0 (1.0–6.0)	3.0 (1.0–5.0)	3.0 (1.0–5.0)	p = 0.49*
Pre-SRT PSA (ng/mL), median (IQR) (n = 163)	0.9 (0.5–1.7)	1.0 (0.5–1.5)	0.6 (0.3–1.0)	0.5 (0.4–0.7)	p = 0.09*
SRT dose (Gy), median (IQR) (n = 171)	68.4 (64.8–68.4)	65.8 (64.8–68.4)	68.4 (64.8–68.4)	64.9 (64.8–68.4)	p = 0.22*
WPRT	11.3%	3.6%	12.5%	8.0%	p = 0.60**
ADT during SRT	12.9%	25.0%	12.5%	12.0%	p = 0.40**
Duration of ADT during SRT (mo), median (IQR)	6.4 (6.0–19.6)	6.2 (3.6–11.1)	6.0 (3.6–7.8)	6.0 (3.0–12.0)	p = 0.43*
Gleason score (n = 171)					
2–6	9.8%	7.1%	3.1%	22.0%	p = 0.04**
7	63.9%	42.9%	53.1%	54.0%	p = 0.30**
8–10	26.2%	50.0%	43.8%	24.0%	p = 0.04**
pT stage (n = 146)					
T1-T2a	10.4%	0.0%	3.3%	4.7%	p = 0.26**
T2b-T2c	18.8%	12.0%	16.7%	16.3%	p = 0.91**
T3-T4	70.8%	88.0%	80.0%	79.1%	p = 0.39**
SVI (n = 171)	21.0%	35.7%	34.4%	26.5%	p = 0.38**
ECE	54.8%	78.6%	75.0%	70.0%	p = 0.07**
SM (n = 169)	38.7%	53.6%	53.3%	36.7%	p = 0.28**
Undetectable PSA nadir post-SRT (n = 170)	69.4%	64.3%	81.30%	45.8%	p = 0.008**
PSA before SADT (ng/mL), median (IQR)	4.3 (2.0–6.7)	2.7 (1.0–8.0)	5.2 (2.4–9.7)	---	p = 0.21*
PSADT after SRT (mo), median (IQR)	6.0 (4.5–9.5)	2.2 (1.5–2.7)	4.1 (1.9–7.3)	27.0 (13.6–47.7)	p < 0.001*
IBF (mo), median (mean)	12.9 (18.1)	11.5 (18.5)	13.6 (13.8)	48.3 (48.7)	p < 0.001*
IBF <18 months	61.3%	67.9%	75.0%	12.0%	p < 0.0001**

*Abbreviations*: PSADT = prostate-specific antigen doubling time, SADT = salvage androgen deprivation therapy, DM = distant metastasis, SRT = salvage radiation therapy, IQR = interquartile range, CCMI = Charlson Comorbidity Index, PSA = prostate-specific antigen, WPRT = whole-pelvic radiation therapy, ADT = androgen deprivation therapy, pT stage = pathologic T stage, SVI = seminal vesicle invasion, ECE = extracapsular extension, SM = presence of any positive surgical margin, IBF = interval to biochemical failure, *Analysis of variance, **Chi-square.

### Univariate analysis

**Figure 1:**
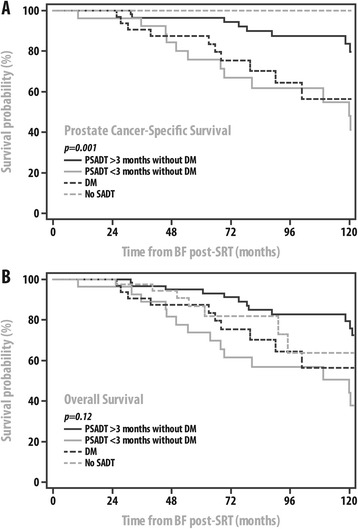
**Kaplan Meier plot of prostate cancer specific survival and overall survival after biochemical failure for patients treated with salvage radiotherapy. A** Displays prostate cancer-specific survival as a function of no use of salvage ADT (SADT) or salvage ADT started after distant metastasis (DM), or before distant metastasis but stratified by short (<3 months) or long (>3 months) PSA doubling time (PSADT) while B shows overall survival based upon the same stratifications. **B**: Kaplan Meier Plot of the Time from BF to the Start of Salvage Androgen Deprivation Therapy (SADT). Patients are Stratified by their clinical status at the time of receiving (SADT) where some had already experienced distant metastasis when SADT was started (DM) while others had no DM but were stratified by short (<3 months) or long (>3 months) PSA doubling time (PSADT).

**Figure 2:**
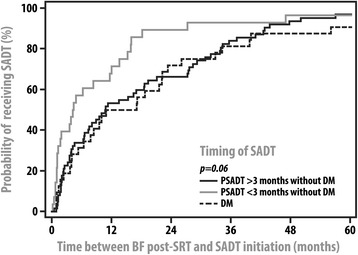
**Time between BF post-SRT and the start of SADT stratified by patient groups 1–3.** Abbreviations: SADT = salvage androgen deprivation therapy, BF = biochemical failure, PSADT = prostate specific-antigen doubling time, SRT = salvage radiation therapy.

Kaplan-Meier survival curves displaying the prostate-cancer specific survival and overall survival of groups 1–4 are displayed in Figure 1. PCSM significantly differed among the four groups (p = 0.001) with 5-year rates of PCSM being 4%, 24%, 13%, and 0% for groups 1–4, respectively; however, OM was not significantly different among the four subsets of patients. There was no statistically significant difference in PCSM or OM when comparing groups 1 and 4 or groups 2 and 3. Of note, patients categorized as having more advanced disease at initiation of SADT were at statistically significantly increased risk for PCSM (HR:2.8, 95% CI: 1.4–5.6, p = 0.005) and OM (HR:1.9, 95% CI: 1.0–3.5, p = 0.04) when compared to those with less advanced disease at initiation of SADT. In addition, a Kaplan-Meier curve depicting the amount of time between BF and SADT initiation stratified by groups 1–3 is displayed in Figure 2. Patients in group 2 were found to receive SADT significantly more quickly than patients in group 1 (p = 0.03).

When using a univariate Cox proportional hazards regression including groups 1–3, PSADT at initiation of SADT as a continuous variable was found to be significantly prognostic for PCSM (hazard ratio [HR]:0.8, 95% confidence interval [CI]: 0.7–0.9, p = 0.0009) and OM (HR:0.9, 95% CI: 0.8–0.96, p = 0.01). PSA level immediately before starting SADT was also significantly predictive of PCSM (HR:1.02, 95% CI: 1.01–1.03, p = 0.002) and OM (HR:1.02, 95% CI: 1.01–1.03, p = 0.002) on univariate Cox proportional hazards regression. C-indices were calculated for pre-SADT PSADT as a continuous variable given its strong prognostic ability and found to be 0.74 for PCSM and 0.71 for OM.

### Multivariate analysis

Multivariate analysis including groups 1–3 was performed while controlling for pre-SADT PSA, pre-SADT PSADT <3 months, DM at SADT initiation, achievement of an undetectable PSA nadir post-SRT, total Gleason score, seminal vesicle invasion, extracapsular extension, presence of any positive surgical margin, use of ADT during SRT, start date of SRT, Charlson Comorbidity Index at the time of SRT [20], and interval to biochemical failure (IBF) <18 months following the start of SRT [19]. Table 2 displays the results of a Cox proportional hazards model including these covariates and shows that a PSADT <3 months prior to initiation of SADT was the most significant predictor of PCSM (HR:4.2, 95% CI: 1.6–11.1, p = 0.004) and only significant predictor of OM (HR:2.9, 95% CI: 1.3–6.7, p = 0.01). Since there is debate about which PSADT cut-point optimizes its prognostic ability for poor outcomes, the same multivariate analysis was performed while including pre-SADT PSADT as a continuous rather than a categorical variable. Pre-SADT PSADT as a continuous variable was again the most significant predictor of PCSM (HR:0.7, 95% CI: 0.6–0.9, p = 0.01) with an interval to biochemical failure <18 months also being predictive for this endpoint (HR:4.8, 95% CI: 1.1–19.9, p = 0.03). On the other hand, PSA prior to initiation of SADT was the only significant predictor of OM when including pre-SADT PSADT as a continuous variable (HR:1.0, 95% CI: 1.0–1.0, p = 0.04).

**Table 2:** **Multivariate analysis**

	**Prostate cancer-specific mortality**		**Overall mortality**	
**Variable**	**HR (95% CI)**	***P***	**HR (95% CI)**	***P***
PSA before SADT	1.0 (1.0–1.0)	0.06	1.0 (1.0–1.0)	0.06
Pre-SADT PSADT <3 months	4.2 (1.6–11.1)	0.004	2.9 (1.3–6.7)	0.01
DM at SADT initiation	1.1 (0.4–3.0)	0.9	0.9 (0.4–2.4)	0.9
Undetectable PSA nadir post-SRT	0.7 (0.2–2.9)	0.7	0.6 (0.2–1.9)	0.4
Total Gleason score	1.2 (0.7–2.1)	0.5	1.0 (0.6–1.7)	1.0
SVI	1.7 (0.6–5.1)	0.3	1.8 (0.7–4.6)	0.2
ECE	0.5 (0.1–2.0)	0.4	0.8 (0.3–2.4)	0.7
SM	1.5 (0.5–4.6)	0.5	1.0 (0.4–2.7)	1.0
ADT during SRT	1.1 (0.3–4.2)	0.9	1.6 (0.5–5.1)	0.4
Start date of SRT	1.0 (1.0–1.0)	0.4	1.0 (1.0–1.0)	0.1
CCMI	1.2 (0.8–1.9)	0.4	1.0 (0.7–1.5)	0.8
IBF <18 months	5.8 (1.3–24.1)	0.02	2.7 (0.9–7.9)	0.07

*Abbreviations*: HR = hazards ratio, CI = confidence interval, PSA = prostate-specific antigen, SADT = salvage androgen deprivation therapy, DM = distant metastasis, SVI = seminal vesicle invasion, ECE = extracapsular extension, SM = presence of any positive surgical margin, ADT = androgen deprivation therapy, SRT = salvage radiation therapy, CCMI = Charlson Comorbidity Index, IBF = interval to biochemical failure.

## Discussion

We performed a single-institution, retrospective review examining prognostic factors for PCSM and OM for men experiencing BF after SRT. These data are limited by selection bias and their non-randomized nature. Despite these limitations, they suggest a decreased risk of PCSM and OM associated with the initiation of SADT while patients have a PSADT >3 months in the absence of DM following BF post-SRT. Whether the relationship between a longer PSADT at SADT initiation and improved clinical outcomes is cause and effect or simply an association observed in this cohort is unknown. However, it does not seem unreasonable that initiating SADT while patients have a lower disease burden or less aggressive clinical characteristics would associate with improved clinical outcomes as this is in accordance with a recent meta-analysis of randomized trials performed in those with advanced prostate cancer [3]. Our findings are the first to indicate that less advanced recurrent disease at the time of SADT initiation post-SRT may be beneficial and, to our knowledge, no other similar retrospective studies in the post-SRT setting have been completed to date. Moreover, prospective trials demonstrating a survival benefit associated with the initiation of SADT early in the disease course for patients experiencing BF alone following RP, definitive RT, or SRT are lacking [4,6].

Of note, our results do not support initiation of SADT in all patients with less advanced disease following BF post-SRT. The lack of a significant difference in PCSM and OM between patients receiving SADT with a low disease burden (group 1) and patients not receiving SADT during follow-up (group 4) suggests that there is a subset of patients for whom initiating SADT while they have less advanced disease may not be beneficial. Given the very long PSADTs and long median IBF for patients in group 4, it is likely that patients experiencing late BF with a gradual, slow rise in PSA may not be reasonable candidates for initiation of SADT. Freedland *et al.* discovered that patients with PSADTs >15 months post-RP were at very low risk for PCSM, confirming that initiating SADT while disease burden is low may not be clinically advantageous for patients with especially long PSADTs [14]. Supporting this possibility is the fact that, like Freedland *et al.*, no patient in the currently analyzed cohort with a PSADT >15 months experienced PCSM. Therefore, it may be reasonable to initially observe patients with very long PSADTs after BF post-SRT and reconsider starting SADT should these patients go on to develop shortened PSADTs during follow-up. On the other hand, for patients with PSADTs that are short enough to put them at a less than negligible risk for PCSM, starting SADT prior to the development of metastasis or a PSADT <3 months is associated with a decreased risk of PCSM and OM based on our findings.

Interestingly, we demonstrated that patients without DM who do not receive SADT until they have a PSADT <3 months have a risk of PCSM and OM comparable to those who start SADT with known DM, which is consistent with similar findings in the post-definitive RT setting [9]. Therefore, patients without DM who do not receive SADT until they have developed short PSADTs may already harbor disease that is sufficiently aggressive to lessen SADT’s therapeutic benefits. Additionally, among patients without DM prior to starting SADT, those with a pre-SADT PSADT <3 months were found to be at a significantly increased risk for PCSM and OM despite having received SADT more quickly than patients with longer PSADTs. Thus, close monitoring of PSADT following BF post-SRT may be warranted to identify even small windows of opportunity to start SADT before the development of a short PSADT.

In our analysis, PSADT before the initiation of SADT was shown to be strongly prognostic of both PCSM and OM on univariate and multivariate analysis. The c-index value of 0.74 for PCSM and 0.71 for OM is further evidence of the prognostic utility of PSADT prior to starting SADT. For comparison purposes, the Stephenson nomogram, which predicts the likelihood of developing BF post-SRT based on eleven clinical and pathologic variables, had a c-index value of 0.69 in the dataset from which the nomogram was derived [1] and a c-index of 0.71 in the dataset from which our presented data was extracted. The fact that PSADT prior to the initiation of SADT demonstrates similar predictive accuracy for the development of PCSM and OM following BF post-SRT highlights its clinical importance.

Our findings contribute to a growing body of work identifying the potential benefits of initiating SADT while patients have less advanced disease. Previous studies examining the benefits of initiating SADT early in the disease course for patients experiencing BF without metastatic disease are solely retrospective and performed in the post-RP or post-definitive RT settings [7-11,21]. In one such study, Moul *et al.* demonstrated the ability of SADT initiation to delay the development of metastasis in high-risk patients with PSA-only failure post-RP [21]. Several other retrospective studies have shown improved overall survival when SADT is started early in the course of recurrent disease following BF post-definitive RT [7-11]. Our results are similar to those reported by Mydin *et al.* who were able to show that patients with a pre-SADT PSA ≤10 ng/mL in the absence of DM had improved overall survival post-definitive RT in a secondary analysis of Irish Clinical Oncology Research Group 97–01 [7]. However, PSADT prior to the initiation of SADT was not prognostic for overall survival on multivariate analysis while timing of SADT administration based on PSA level and presence or absence of DM at initiation of SADT was prognostic for this endpoint [7]. In contrast, Tenenholz *et al.* have previously shown PSADT prior to the initiation of SADT to be the most powerful predictor of overall survival and disease-specific survival in a retrospective analysis of patients experiencing BF post-definitive RT [9]. Thus, findings regarding whether PSA or PSADT prior to starting SADT is a superior prognostic factor for outcomes post-definitive RT are mixed. Our results suggest that a PSADT <3 months prior to starting SADT is more prognostic for risk of PCSM and OM than the pre-SADT PSA level in the post-SRT setting. This is not surprising considering that patients post-prostatectomy should have uniformly low PSA levels, and those harboring aggressive disease may develop short PSADTs even before their PSA levels have a chance to become markedly elevated once again. Another prognostic factor that has been utilized to assess clinical risk is the IBF after the start of SRT [19], which likely has some overlap with PSADT in that they may be measuring similar biological processes. In the current analysis, it appeared that a very short PSADT still added significant clinical value when including both of these metrics in our multivariate Cox proportional hazards models.

As previously discussed, the inherent limitations to this study secondary to its retrospective design warrant further validation of our findings. A second limitation of this analysis is that we were unable to assess toxicity related to the use of SADT. The potential negative health effects of ADT and ADT’s adverse effects on quality of life must be considered when contemplating the appropriate clinical context for its initiation. For instance, long-term use of ADT is known to increase the risk of experiencing a skeletal fracture and is associated with a higher risk of developing type 2 diabetes mellitus [22]. Additionally, ADT puts one at a higher risk for mortality secondary to cardiovascular disease and is known to cause vasomotor hot flashes and sexual side effects that can be associated with a lower quality of life [22,23]. Thus, the administration of ADT is not benign and the decision to initiate this treatment should be made after carefully considering the potential risks and benefits of this therapeutic intervention. Nevertheless, the possibility that survival may be prolonged by initiating SADT when patients have less advanced recurrent disease post-SRT make this approach an attractive option, especially in high-risk patients such as ours who have exhausted localized treatment options [6].

## Conclusions

Overall, our results suggest that initiating SADT while patients have less advanced disease following BF post-SRT may be a sensible treatment option associated with a decreased risk of PCSM and OM. However, observation may be a reasonable alternative for patients with especially long PSADTs given their very low risk of experiencing PCSM. Furthermore, a PSADT <3 months before the initiation of SADT appears to significantly predict an increased risk of PCSM and OM for patients with biochemically recurrent disease following SRT. Future prospective studies examining the optimal clinical context for starting SADT post-SRT should consider using the development of a short PSADT as a condition for initiation of SADT given the significant ability of pre-SADT PSADT <3 months to predict mortality in our analysis.

## Abbreviations

ADT: Androgen deprivation therapy
BF: Biochemical failure
c-index: Concordance index
CCMI: Charlson Comorbidity Index
CI: Confidence interval
DM: Distant metastasis
ECE: Extracapsular extension
HR: Hazard ratio
IBF: Interval to biochemical failure
IQR: Interquartile range
OM: Overall mortality
PCSM: Prostate cancer-specific mortality
PSA: Prostate-specific antigen
PSADT: Prostate-specific antigen doubling time
pT stage: Pathologic T stage
RP: Radical prostatectomy
RT: Radiotherapy
SADT: Salvage androgen deprivation therapy
SM: Any positive surgical margin
SRT: Salvage radiation therapy
SVI: Seminal vesicle invasion
WPRT: Whole-pelvic radiation therapy

## References

[1] Stephenson AJ, Scardino PT, Kattan MW, Pisansky TM, Slawin KM, Klein EA, Anscher MS, Michalski JM, Sandler HM, Lin DW, Forman JD, Zelefsky MJ, Kestin LL, Roehrborn CG, Catton CN, DeWeese TL, Liauw SL, Valicenti RK, Kuban DA, Pollack A (2007). Predicting the outcome of salvage radiation therapy for recurrent prostate cancer after radical prostatectomy. J Clin Oncol.

[2] Huggins C, Hodges CV (1941). Studies on prostatic cancer II: the effects of castration on advanced carcinoma of the prostate gland. Arch Surg.

[3] Nair B, Wilt T, MacDonald R, Rutks I (2002). Early versus deferred androgen suppression in the treatment of advanced prostatic cancer. Cochrane Database Syst Rev.

[4] Ryan CJ, Small EJ (2005). Early versus delayed androgen deprivation for prostate cancer: new fuel for an old debate. J Clin Oncol.

[5] Bruce JY, Lang JM, McNeel DG, Liu G (2012). Current controversies in the management of biochemical failure in prostate cancer. Clin Adv Hematol Oncol.

[6] Moul JW, Bañez LL, Freeland SJ (2007). Rising PSA in nonmetastatic prostate cancer. Oncology.

[7] Mydin AR, Dunne MT, Finn MA, Armstrong JG (2013). Early salvage hormonal therapy for biochemical failure improved survival in prostate cancer patients after neoadjuvant hormonal therapy plus radiation therapy–a secondary analysis of irish clinical oncology research group 97–01. Int J Radiat Oncol Biol Phys.

[8] D’Amico AV, Cote K, Loffredo M, Renshaw AA, Schultz D (2002). Determinants of prostate cancer-specific survival after radiation therapy for patients with clinically localized prostate cancer. J Clin Oncol.

[9] Tenenholz TC, Shields C, Ramesh VR, Tercilla O, Hagan MP (2007). Survival benefit for early hormone ablation in biochemically recurrent prostate cancer. Urol Oncol.

[10] Shipley WU, DeSilvio M, Pilepich MV, Roach M, Wolkov HB, Sause WT, Rubin P, Lawton CA (2006). Early initiation of salvage hormone therapy influences survival in patients who failed initial radiation for locally advanced prostate cancer: a secondary analysis of RTOG protocol 86–10. Int J Radiat Oncol Biol Phys.

[11] Souhami L, Bae K, Pilepich M, Sandler H (2010). Timing of salvage hormonal therapy in prostate cancer patients with unfavorable prognosis treated with radiotherapy: a secondary analysis of Radiation Therapy Oncology Group 85–31. Int J Radiat Oncol Biol Phys.

[12] Cookson MS, Aus G, Burnett AL, Canby-Hagino ED, D’Amico AV, Dmochowski RR, Eton DT, Forman JD, Goldenberg SL, Hernandez J, Higano CS, Kraus SR, Moul JW, Tangen C, Thrasher JB, Thompson I (2007). Variation in the definition of biochemical recurrence in patients treated for localized prostate cancer: the American Urological Association Prostate Guidelines for Localized Prostate Cancer Update Panel report and recommendations for a standard in the reporting of surgical outcomes. J Urol.

[13] Pound CR, Partin AW, Eisenberger MA, Chan DW, Pearson JD, Walsh PC (1999). Natural history of progression after PSA elevation following radical prostatectomy. JAMA.

[14] Freedland SJ, Humphreys EB, Mangold LA, Eisenberger M, Dorey FJ, Walsh PC, Partin AW (2005). Risk of prostate cancer-specific mortality following biochemical recurrence after radical prostatectomy. JAMA.

[15] Zhou P, Chen MH, McLeod D, Carroll PR, Moul JW, D’Amico AV (2005). Predictors of prostate cancer-specific mortality after radical prostatectomy or radiation therapy. J Clin Oncol.

[16] D’Amico AV, Moul JW, Carroll PR, Sun L, Lubeck D, Chen MH (2003). Surrogate end point for prostate cancer-specific mortality after radical prostatectomy or radiation therapy. J Natl Cancer Inst.

[17] Jackson WC, Johnson SB, Li D, Foster C, Foster B, Song Y, Schipper M, Shilkrut M, Sandler HM, Morgan TM, Palapattu GS, Hamstra DA, Feng FY (2013). A prostate-specific antigen doubling time of <6 months is prognostic for metastasis and prostate cancer-specific death for patients receiving salvage radiation therapy post radical prostatectomy. Radiat Oncol.

[18] Harrell FE, Califf RM, Pryor DB, Lee KL, Rosati RA (1982). Evaluating the yield of medical tests. JAMA.

[19] Johnson S, Jackson W, Li D, Song Y, Foster C, Foster B, Zhou J, Vainshtein J, Feng F, Hamstra D (2013). The interval to biochemical failure is prognostic for metastasis, prostate cancer-specific mortality, and overall mortality after salvage radiation therapy for prostate cancer. Int J Radiat Biol Oncol Phys.

[20] Charlson ME, Pompei P, Ales KL, MacKenzie CR (1987). A new method of classifying prognostic comorbidity in longitudinal studies: development and validation. J Chronic Dis.

[21] Moul JW, Wu H, Sun L, McLeod DG, Amling C, Donahue T, Kusuda L, Sexton W, O’Reilly K, Hernandez J, Chung A, Soderdahl D (2004). Early versus delayed hormonal therapy for prostate specific antigen only recurrence of prostate cancer after radical prostatectomy. J Urol.

[22] Taylor LG, Canfield SE, Du XL (2009). Review of major adverse effects of androgen-deprivation therapy in men with prostate cancer. Cancer.

[23] Schwandt A, Garcia JA (2009). Complications of androgen deprivation therapy in prostate cancer. Curr Opin Urol.

